# Women’s perceived partner support during the perinatal and early childhood period: changes over time for women with and without Major Depressive Disorder

**DOI:** 10.1007/s00737-026-01706-1

**Published:** 2026-05-07

**Authors:** Irene Bobevski, Karen Wynter, Philip Boyce, Megan Galbally

**Affiliations:** 1 Department of Psychiatry, School of Clinical Sciences, Monash University, Melbourne, Australia; 2 School of Nursing and Midwifery, Deakin University, Geelong, Australia; 3 Specialty of Psychiatry, Westmead Institute of Medical Research, The University of Sydney, Sydney, Australia

**Keywords:** Social support, Partner, Pregnancy, Postpartum, Depression

## Abstract

**Background:**

Partner support is both an important protective and risk factor for women’s mental health perinatally. Although there is likely a bidirectional relationship between support and mental health, a research gap exists in understanding changes in women’s experience of partner support over pregnancy and early childhood, and whether this differs for women with Major Depressive Disorder (MDD). This study examines whether women diagnosed with MDD antenatally are at increased risk of deteriorating partner support over the perinatal period, after accounting for demographic effects, ongoing depressive symptoms, stressful life events, and attachment orientation.

**Methods:**

731 women recruited into a longitudinal pregnancy cohort study, the Mercy Pregnancy Emotional Wellbeing Study, were included, of whom 124 were diagnosed with MDD first trimester using the Structured Clinical Interview for the DSM (SCID). Perceived partner support was measured with the Social Support Effectiveness Questionnaire (SSEQ) in third trimester, 6 and 12 months, and 4 years postpartum. Partner support changes over time were analysed with mixed effects modelling.

**Results:**

There was an overall small but significant decline in partner support over time for all women. However, this decline was larger for women with MDD between 12 months and 4 years postpartum. Ongoing depressive symptoms, stressful life events, and insecure attachment orientation contributed to perceptions of lower partner support.

**Conclusions:**

The perinatal and early childhood period poses an increased risk for the partner relationship for all women, but this risk is increased for women with MDD. This knowledge could be translated into identifying vulnerable women and offering appropriate interventions.

## Introduction

The transition to parenthood can be a time of joy, but it is also one of the most demanding and challenging life stages. It brings change to roles and relationships, requiring re-organisation and re-negotiation of individuals’ roles, as well as intimate partner relationship and family roles (Figueiredo et al. 2018).

During this transition, a poor-quality interpersonal relationship is a risk factor for the development of postnatal depression (for example: Boyce and Hickey 2005; Boyce et al. 1991), while positive partner relationship, especially perceived partner support, is a very important protective and potentially modifiable factor (Pilkington et al. 2015a, b). Research evidence has clearly shown that a good quality partner relationship is protective against women’s perinatal mental health problems (Bedaso et al. 2021, 2023; Figueiredo et al. 2008; Galbally et al. 2019; Whisman et al. 2011) and is linked to more positive child outcomes (Lähdepuro et al. 2024; Ray et al. 2024; Schuijers et al. 2024; Stapleton et al. 2012). In our previous research Galbally et al. (2019) found that partner support had a protective role against parenting distress for women with no history of childhood abuse and low depressive symptoms, but not for women with trauma history and high depressive symptoms.

A good understanding of how the partner relationship changes over time and how it is related to women’s perinatal mental health is essential for elucidating risk and protective factors during this life stage. This is best achieved through longitudinal research. Many of the existing longitudinal studies have focused on how the quality of the intimate partner relationship affects a woman’s mental health. However, this is a bi-directional relationship: a good quality relationship and support can help women to adjust and cope with the stresses of parenthood, and be protective against mental health problems. Conversely, women’s experiences, the external environment, and caring for a newborn can affect the intimate relationship (Najman et al. 2014; Nakamura et al. 2020).

Numerous studies have found relationship quality to be a protective factor for women’s perinatal mental health problems, as described earlier (Bedaso et al. 2021, 2023; Figueiredo et al. 2008; Galbally et al. 2019; Whisman et al. 2011). Although fewer studies have examined the effect of mental health problems on the relationship, a consistent finding is that depressive and/or anxiety symptoms contribute to deterioration in the marital relationship (Bower et al. 2013; Parfitt and Ayers 2014). Large longitudinal studies have found a bi-directional association between poor marital quality and depressive symptoms over time, from pregnancy to 2 years (Salmela-Aro et al. 2006) and even 21 years postpartum (Najman et al. 2014). Nevertheless, one study (Figueiredo et al. 2018) found that anxiety and depression symptoms had no moderating effect on trajectories of positive and negative couple interactions, although mothers and fathers with high negative interactions had a greater increase in depressive symptoms from 3 to 30 months postpartum. These differing findings may be due to studies measuring different aspects of relationship quality.

Generally, studies have shown evidence of a declining relationship satisfaction over time, but some have found a stable trajectory. Longitudinal studies, examining the trajectory of the quality of partner relationship over the perinatal period across different countries, have found a small to moderate decline in couple relationship satisfaction from pregnancy to postpartum (Kluwer 2010; Mitnick et al. 2009). A sharp decline has been shown up to 1 year postpartum (Lawrence et al. 2008; Twenge et al. 2003), but also up to early childhood (Kohn et al. 2012), and even 14.5 years after the birth of a baby (Keizer and Schenk 2012). A 5-year longitudinal study (Kluwer and Johnson 2007) found a linear decrease in relationship quality from pregnancy to 15 months postpartum, but then relationship quality scores levelled off to 4 years postpartum. Conversely, some studies examining subgroups with varying patterns of relationship changes, have found a stable relationship satisfaction trajectory for substantial proportions of couples, from 47% (Leonhardt et al. 2022) to 93% (Kingsbury et al. 2023).

Some studies have compared trajectories of relationship satisfaction for different types of couples and families from before birth to postpartum. A steeper relationship satisfaction decline has been shown in first- than second-time parents (Canário and Figueiredo 2016), and for couples with initial lower relationship satisfaction (Don and Mickelson 2014). A larger and more sudden relationship decline has been found in childbearing couples compared to childless couples (Doss et al. 2009; Kluwer 2010; Lawrence et al. 2008). A meta analytic study (Twenge et al. 2003) found a cohort effect, with more recent generations of parents reporting greater relationship decline, perhaps due to changes in gender roles over time.

The associations between partner support or relationship satisfaction and women’s demographic characteristics and attachment orientation have also been examined. Women’s low socio-economic status (Nakamura et al. 2020), no employment during early parenthood (Hetherington et al. 2020; Keizer et al. 2010), and minority ethnicity (Hetherington et al. 2020) tend to contribute to lower partner or overall social support perinatally. Women’s anxious or avoidant attachment orientation in close relationships have also been associated with a decline in relationship satisfaction during the perinatal period (Kohn et al. 2012; Leonhardt et al. 2022; Rholes et al. 2014).

Most longitudinal studies have focused on overall relationship quality and relatively few have examined specific aspects of partner support during the perinatal period. Focusing on specific aspects of support, such as emotional and practical, will enhance the translation of this information into targeted preventions and interventions (Pilkington et al. 2015a, b). Both emotional and practical partner support have been established as important protective factors against women’s perinatal menta health symptoms (Pilkington et al. 2015a, b). However, little is known about how they change over time during the transition to parenthood. During the perinatal period partner support is especially important compared to support from other sources and uniquely contributes to women’s perinatal adjustment (Kroelinger and Oths 2000; Pajulo et al. 2001; Rini 2001). In addition to the quantity of received support, women’s perceived effectiveness of received support is an important determinant of whether the support will have beneficial effects for their wellbeing (Rini 2001).

There is also a lack of longitudinal studies examining the perinatal trajectory of partner support for women with diagnosed mental health disorders, with most studies focusing only on depressive symptoms. Major Depressive Disorder is prevalent and causes significant burden for women perinatally. It is important to understand if women with Major Depressive Disorder are at an increased risk for insufficient or deteriorating partner support over the perinatal period.

To address these identified gaps in research this study aims to examine first, how women’s perceptions of the effectiveness of emotional and practical partner support change from late pregnancy, to 6 and 12 months postpartum, and then when the child is 4 years of age.

Secondly, we aim to examine whether these changes over time differ between women with, and without, Major Depressive Disorder (MDD). Finally, we aim to investigate whether socioeconomic factors, stressful life events, attachment orientation in close relationships.

## Methods

### Sample

Participant for this longitudinal study were women initially recruited at less than 20 weeks of pregnancy. The MPEWS is an Australian prospective pregnancy cohort study of women recruited at less than 20 weeks of pregnancy and followed up overtime. Women were recruited from Victoria and Western Australia.

For the purposes of this study we used a nested sample from the Mercy Pregnancy and Emotional Wellbeing Study (MPEWS). We used data collected at less than 20 weeks pregnancy (recruitment), third trimester, 6 months postpartum, 12 months postpartum, and when the child was 4 years old. The MPEWS used a selected cohort design to oversample women diagnosed with MDD at recruitment. Women were recruited into MPEWS through the antenatal booking process in general hospitals. In order to over-sample women with MDD, women were also recruited through a perinatal mental health clinic. Further details of MPEWS are described in the published study protocol (Galbally et al. 2017). 

### Inclusion criteria

Since this study focused on changes in perceived partner support over time, only women with a continuous relationship with the same partner from first trimester to when the child is 4 years of age were included. 

### Ethics

The Ethics Committees of the participating institutions approved this study and all participants provided informed, written consent.

### Measures

#### Demographic and socioeconomic characteristics

MPEWS collected data on maternal age, university education, employment, ethnicity of the mother and father, and parity at recruitment. Reliable data on parity was only available at recruitment, and therefore the analysis could not account for women giving birth to additional children within the four years timespan of the study. Data on relationship status was collected at recruitment and at the follow up time points.

#### Maternal Major Depressive Disorder and depressive symptoms

At recruitment (less than 20 weeks gestation), the Structured Clinical Interview for DSM-IV (SCID-IV) was administered (First et al. 1997) to diagnose MDD. Depressive symptoms were measured using the Edinburgh Postnatal Depression Scale (EPDS) (Cox et al. 1987). For this study EPDS data at third trimester, 6 and 12 months, and 4 years postpartum was used. The EPDS has been validated for use with Australian women during the perinatal period (Boyce et al. 1993). The EPDS consists of 10 items measuring depressive symptoms on a 4-point scale. Total scores range from 0 to 30, with higher scores indicating more severe depressive symptoms. Scores of 13 or higher are typically used as an indication of a depressive episode (Cox et al. 1987).

#### Social support effectiveness questionnaire

Social support was measured using two, of the three, subscales from the Social Support Effectiveness Questionnaire (SSEQ) (Rini 2001). These two scales, ‘Help with tasks and responsibilities’ and ‘Emotional support’, each consist of 5 items, asking the respondent to evaluate the quantity and effectiveness of the support provided by her partner on a 5-point scale. Subscale scores range from 1 to 25, with higher scores indicating higher perceived support. The SSEQ asks the extent to which the partner’s attempts at support meets the needs of the respondent, including the ease with which the support is obtained, and the match between what is needed and what is provided. Women’s perceived effectiveness of received support is an important determinant of whether the support will have beneficial effects for their wellbeing (Rini 2001). The SSEQ has been shown to be associated with maternal and infant distress in the postpartum period, and to relate to facets of maternal attachment orientation (Stapleton et al. 2012). The SSEQ was administered to women at third trimester, six, 12 months, and four years postpartum. The third SSEQ scale about informational support was not included in the MPEWS study, since emotional and practical support were considered as central to the relationship, while informational support during pregnancy and postpartum is often provided by external sources, and also to reduce the burden to participants.

#### Stressful life events scale (SLES)

Stressful life events were assessed using a perinatally adapted Stressful Life Events Scale, which includes 24 common stressful life events (Brown et al. 2011b). Items specifically for the perinatal period were selected from the Stressful Life Events Scale (Whitehead et al. 2003), and adapted and extended by Brown and colleagues. The measure includes items such as whether the respondent had a major illness or injury, got separated or divorced, lost her job, or was humiliated or emotionally abused by her partner. The items were adapted from Brown et al. (2011a). A count of stressful life events was calculated separately for third trimester, six and 12 months, and 4 years postpartum.

#### Adult attachment orientation in close relationships

Adult attachment relationship orientation was evaluated using the Experiences in Close Relationships – Short Form (ECR-SF) (Wei et al. 2007), a 12-item self-report measure which yields two aspects of adult attachment orientation – avoidance and anxiety. Avoidance involves fear of dependence and interpersonal intimacy, excessive need for self-reliance, and reluctance to self-disclose. Anxiety involves fear of rejection or abandonment, excessive need for approval, and distress when one’s partner is unavailable or unresponsive (Wei et al. 2007). Items are measured on a 7-point scale, with scores for each subscale ranging from 1 to 42, with higher scores indicating a higher level of anxious or avoidant attachment orientation. The ECR-CF was administered at first trimester and third trimester of pregnancy and 6 months postpartum.

### Statistical analysis

Descriptive statistics of the sociodemographic characteristics of the sample were examined by diagnosis of MDD, using χ^2^ tests for frequencies, t-tests for continuous, and Mann-Whitney tests for non-normally distributed continuous variables.

To investigate the change in women’s perceptions of partner support over time, mixed effect models were used. Random effects for the intercept and for time were estimated. Mixed models with random effects for the intercept and for time allow for individual variation in addition to mean variation over time. These models are also appropriate for handling missing data, using Maximum Likelihood estimation methods with all available longitudinal data to provide valid inferences for parameters without requiring complete cases at each time points (Rabe-Hesketh and Skrondal 2008). As a sensitivity analysis to investigate if the trajectories over time of participants with missing data may have been significantly different, the mixed models were also run with inverse probability weights for non-completion. Probability weights were estimated through logistic regression, using recruitment characteristics which were significantly different between completers and non-completers to predict non-completion. This method gives more weight to individuals resembling those on characteristics predictive of missingness who did not complete the survey to establish if their trajectories over time may have been different.

Separate models were fitted for emotional support, support with tasks and responsibilities, and overall support (the total score of the two subscales). For each of these outcomes, a model with an interaction term between diagnosis of MDD and time was tested, to determine if perceived partner support of women with and without MDD differed significantly over the time points of the study. A statistically significant interaction term would indicate that for women with and without MDD perceptions of partner support changed differently over time.

All models were controlled for maternal age, university education (yes vs. no), employment, parity, maternal and partner ethnicity, depressive symptoms over time, stressful life events, and attachment orientation in close relationships, based on the literature. Depressive symptoms and stressful life events were modelled as time varying variables. The ECR measures of anxious and avoidant attachment orientation were averaged over time from early pregnancy to 6 months postpartum.

All analysis was conducted with Stata 18 (StataCorp 2021).

## Results

### Sample

Of the total 841 women participating in the MPEWS study, 731 (82%) were in a continuing relationship with the same partner from first trimester (recruitment) to 4 years postpartum, 89 (10%) were in a relationship at recruitment which did not continue for the duration of the study, 26 (3%) were not in a relationship, and 41 (5%) had missing relationship data. Only the 731 women who were in a continuing relationship from first trimester to four years postpartum were included in the analysis of the current study. 

### Demographics and descriptive characteristics

Demographics and descriptive characteristics of the women are presented in Table 1. The mean age of the women was 32.0 (SD 4.0) and 31.3 (SD 5.2) years for women without and with MDD, respectively. Women with MDD were significantly less likely to be university educated (45%) and employed (75%) at recruitment than women without MDD (63% and 84% respectively), see table. There were no significant differences in other demographic characteristics.

**Table 1:** Demographics and descriptive characteristics by MDD

	No MDD (*n*=607)		MDD (*n*=124)		*p*
	**n**	**%**	**n**	**%**	
University educated	385	63.4%	56	45.2%	<.001*
Not nulliparous	199	32.9%	50	40.3%	.112
Employed	508	84.0%	93	75.0%	.017*
Mather not of Oceanic/European background	74	12.2%	16	12.9%	.826
Father not of Oceanic/European background	170	28.1%	26	21.0%	.105
	**M**	**SD**	**M**	**SD**	***p***
Maternal age at recruitment	32.0 Range 19-48	4.4	31.3 Range 21-45	5.2	.160
EPDS at recruitment	5.3 Range 0-21	4.3	9.0 Range 0-24	5.4	<.0001*
Maternal anxious attachment orientation in close relationships	19.2 Range 6-39	5.7	21.2 Range 6-37	6.0	.0004*
Maternal avoidant attachment orientation in close relationships	11.6 Range 6-30	4.4	13.5 Range 6-27	5.0	<.0001*
	**Median**	**IQR**	**Median**	**IQR**	***p***
Number of stressful life events at recruitment	0 Range 0-7	0-2	1 Range 0-7	0-3	.0001*

* *p*-value > .05. Abbreviations: *MDD *Major Depressive Disorder, *M *Mean, *SD *Standard Deviation, *IQR *Interquartile Range, *EPDS *Edinburgh Postnatal Depression Scale

### Comparison of the subsample of the current study to the full MPEWS study

Women with a continuing relationship were compared to the rest of the MPEWS sample on recruitment characteristics. The only statistically significant differences were that women with a continuing relationship tended to be slightly younger (31.8 vs. 32.2 years) and less likely to be diagnosed with MDD (17% vs. 26%). 

### MPEWS attrition rate and missing data

At 4 years postpartum 97% of the 841 women had remained in the MPEWS study. However, not all women competed all items and measures within questionnaires at each time point. Of the 731 women with a continuing relationship from recruitment to 4 years postpartum, the SSEQ was completed by 89% of women at third trimester, 74% at 6 months postpartum, 62% at 12 months postpartum, and 58% at 4 years postpartum. Women who did not complete the SSEQ at all four time points of the current study were significantly more likely to be slightly younger (31.2 vs. 32.5 years), multiparous (41% vs. 26%), and of minority ethnicity (15% vs. 9%). They were significantly less likely to be university educated (53% vs. 69%) and employed at recruitment (78% vs. 88%). There was no statistically significant difference in diagnosis of MDD between women who did not complete the SSEQ at all four time points (16%) and those who did (18%). This pattern was the same for missing SSEQ data at each study wave. 

### Results from mixed effects models

**Fig. 1:**
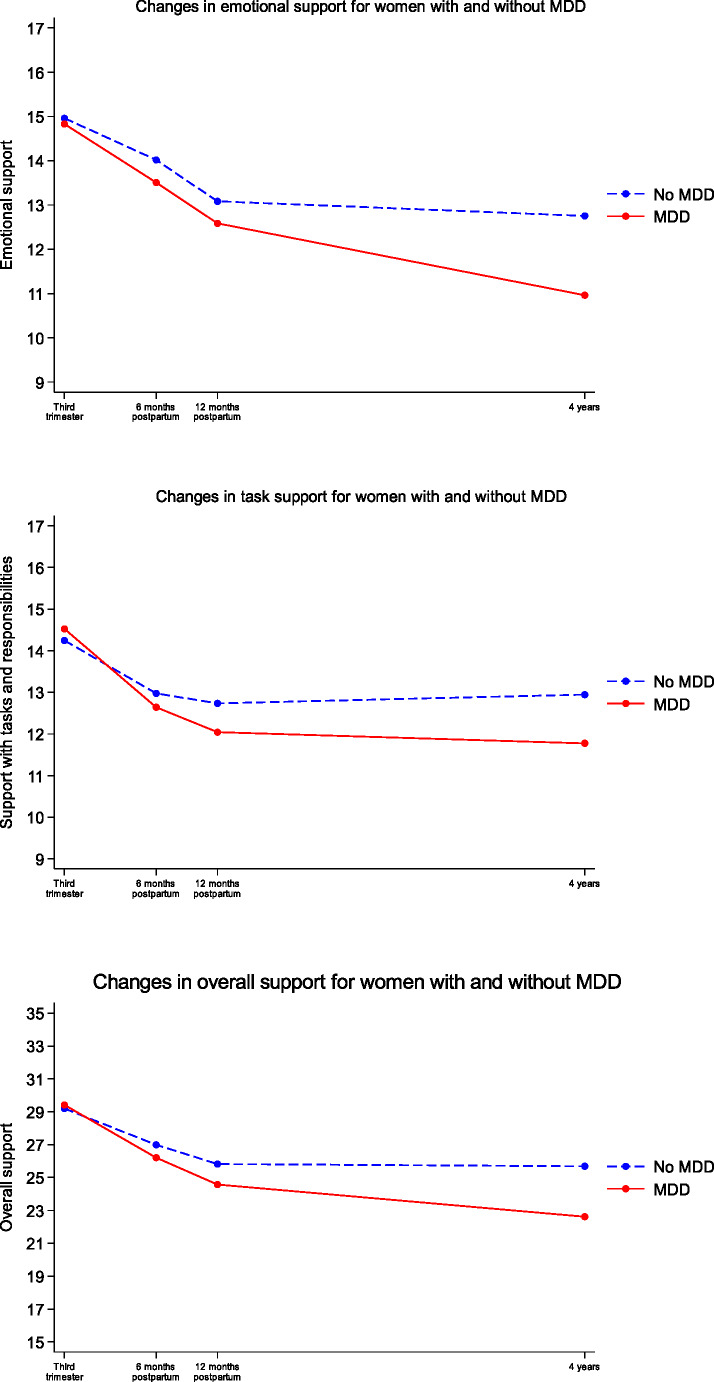
Trajectories of perceived partner support over the perinatal period for women with and without Major Depressive Disorder (MDD)

The results from the mixed effects models are presented in Table 2. Time was modelled as discrete time points (6, 12 months and 4 years postpartum), since it was found to have non-linear trend and since there are only a small number of time points. Plots of the estimated mean trajectories of task, emotional, and overall partner support for women with and without MDD are shown in Fig. 1.

**Table 2:** Mixed effects models for predicting perceived emotional, task, and overall support

		Model 1 with no interactions			Model 2 with interaction term			
**SSEQ Emotional Support**	***b***	**95% CI**		***p***	***b***	**95% CI**		***p***
Time:								
6 months postpartum	-1.01	-1.32	-0.70	<.001*	-0.94	-1.29	-0.60	<.001*
12 months postpartum	-1.94	-2.27	-1.61	<.001*	-1.88	-2.24	-1.52	<.001*
4 years	-2.49	-2.90	-2.09	<.001*	-2.21	-2.64	-1.77	<.001*
MDD	-0.51	-1.15	0.13	.121	-0.13	-0.87	0.61	.732
EPDS	-0.18	-0.21	-0.14	<.001*	-0.18	-0.22	-0.14	<.001*
Maternal age	-0.04	-0.09	0.02	.203	-0.03	-0.09	0.02	.217
University educated	-0.19	-0.71	0.32	.464	-0.19	-0.70	0.33	.474
Parity (not nulliparous)	-0.13	-0.67	0.40	.621	-0.15	-0.68	0.39	.594
Employed	0.08	-0.60	0.75	.828	0.07	-0.61	0.74	.846
Maternal minority ethnicity	-0.53	-1.30	0.25	.183	-0.53	-1.30	0.25	.181
Partner minority ethnicity	0.01	-0.55	0.56	.982	0.01	-0.54	0.57	.969
Number of stressful life events	-0.11	-0.20	-0.02	.016*	-0.11	-0.20	-0.02	.013*
Maternal anxious attachment orientation in close relationships	-0.09	-0.14	-0.04	<.001*	-0.09	-0.14	-0.04	<.001*
Maternal avoidant attachment orientation in close relationships	-0.29	-0.36	-0.23	<.001*	-0.29	-0.36	-0.23	<.001*
Intercept	15.19	14.43	15.94	<.001*	15.13	14.37	15.89	<.001*
*Interaction term*								
Time*MDD:								
6 months postpartum*MDD	-	-	-	-	-0.38	-1.19	0.42	.354
12 months postpartum*MDD	-	-	-	-	-0.37	-1.28	0.54	.429
4 years*MDD	-	-	-	-	-1.66	-2.70	-0.63	.002*
*Random effects parameters*	Estimate	95% CI			Estimate	95% CI		
Variance of the random intercept	6.58	5.64	7.68		6.60	5.66	7.70	
Variance of the random time effects:								
6 months postpartum	0.48	0.02	12.59		0.57	0.04	8.97	
12 months postpartum	1.65	0.59	4.63		1.71	0.63	4.62	
4 years	5.14	3.41	7.73		4.92	3.24	7.47	
**SSEQ Task Support**	***b***	**95% CI**		***p***	***b***	**95% CI**		***p***
Time:								
6 months postpartum	-1.38	-1.70	-1.06	<.001*	-1.27	-1.63	-0.92	<.001*
12 months postpartum	-1.67	-1.98	-1.36	<.001*	-1.51	-1.85	-1.17	<.001*
4 years	-1.55	-1.91	-1.19	<.001*	-1.30	-1.69	-0.91	<.001*
MDD	-0.30	-0.91	0.32	.344	0.28	-0.45	1.01	.451
EPDS	-0.13	-0.17	-0.10	<.001*	-0.14	-0.17	-0.10	<.001*
Maternal age	0.03	-0.02	0.08	.247	0.03	-0.02	0.08	.224
University educated	-0.08	-0.57	0.41	.751	-0.08	-0.57	0.41	.757
Parity (not nulliparous)	0.22	-0.29	0.73	.389	0.21	-0.30	0.72	.419
Employed	-0.05	-0.69	0.60	.885	-0.05	-0.70	0.59	.869
Maternal minority ethnicity	0.03	-0.70	0.77	.929	0.03	-0.71	0.76	.939
Partner minority ethnicity	0.44	-0.09	0.97	.106	0.44	-0.09	0.97	.103
Number of stressful life events	-0.05	-0.14	0.04	.254	-0.05	-0.14	0.03	.217
Maternal anxious attachment orientation in close relationships	-0.04	-0.09	0.00	.055	-0.04	-0.09	0.00	.057
Maternal avoidant attachment orientation in close relationships	-0.26	-0.32	-0.20	<.001*	-0.26	-0.32	-0.20	<.001*
Intercept	14.25	13.52	14.97	<.001*	14.16	13.43	14.88	<.001*
*Interaction Term*								
Time*MDD:								
6 months postpartum*MDD	-		-	-	-0.61	-1.44	0.22	.149
12 months postpartum*MDD	-		-	-	-0.97	-1.82	-0.12	.025*
4 years*MDD	-		-	-	-1.45	-2.37	-0.52	.002*
*Random effects parameters*	Estimate	95% CI			Estimate	95% CI		
Variance of the random intercept	5.91	5.04	6.92		5.92	5.06	6.93	
Variance of the random time effects:								
6 months postpartum	1.08	0.31	3.74		1.10	0.32	3.78	
12 months postpartum	0.00	0.00	0.00		0.00	0.00	0.00	
4 years	1.94	0.87	4.32		1.88	0.82	4.30	
**SSEQ Overall Support**	***b***	**95% CI**		***p***	***b***	**95% CI**		***p***
Time:								
6 months postpartum	-2.38	-2.93	-1.84	<.001*	-2.21	-2.81	-1.61	<.001*
12 months postpartum	-3.62	-4.17	-3.07	<.001*	-3.38	-3.97	-2.78	<.001*
4 years	-4.08	-4.76	-3.40	<.001*	-3.51	-4.25	-2.78	<.001*
MDD	-0.77	-1.89	0.36	.181	0.22	-1.09	1.53	0.745
EPDS	-0.31	-0.37	-0.24	<.001*	-0.31	-0.38	-0.25	<.001*
Maternal age	-0.00	-0.10	0.09	.988	0.00	-0.09	0.10	0.976
University educated	-0.25	-1.15	0.65	.587	-0.24	-1.15	0.66	0.598
Parity (not nulliparous)	0.10	-0.83	1.04	.829	0.08	-0.86	1.01	0.875
Employed	-0.03	-1.22	1.16	.959	-0.05	-1.24	1.14	0.932
Maternal minority ethnicity	-0.47	-1.83	0.88	.495	-0.48	-1.84	0.88	0.488
Partner minority ethnicity	0.50	-0.47	1.48	.312	0.51	-0.47	1.48	0.306
Number of stressful life events	-0.14	-0.30	0.01	.061	-0.15	-0.30	-0.00	0.048
Maternal anxious attachment orientation in close relationships	-0.14	-0.22	-0.06	<.001*	-0.14	-0.22	-0.06	0.001
Maternal avoidant attachment orientation in close relationships	-0.55	-0.66	-0.45	<.001*	-0.55	-0.66	-0.45	<.001*
Intercept	29.45	28.12	30.77	<.001*	29.30	27.97	30.63	<.001*
*Interaction Term*								
Time*MDD:								
6 months postpartum*MDD	-		-	-	-1.00	-2.40	0.40	0.162
12 months postpartum*MDD	-		-	-	-1.47	-2.97	0.04	0.056
4 years*MDD	-		-	-	-3.28	-5.01	-1.56	<.001*
*Random effects parameters*	Estimate		95% CI		Estimate		95% CI	
Variance of the random intercept	20.72	17.79	24.12		20.77	17.84	24.17	
Variance of the random time effects:								
6 months postpartum	1.11	0.02	70.81		1.44	0.06	34.81	
12 months postpartum	1.28	0.03	52.66		1.59	0.08	31.18	
4 years	12.00	7.32	19.67		11.45	6.91	18.97	

* *p*-value > 0.05. Abbreviations: C*I C*onfidence Interval, *SSEQ *Social Support Effectiveness Questionnaire, *MDD *Major Depressive Disorder, *EPDS *Edinburgh Postnatal Depression Scale

Each mixed model was also run with inverse probability weights for non-completion. The results were very similar to the non-weighted models, suggesting that if respondents with similar recruitment characteristics to those with missing data were included in the analysis the results would not have been substantially altered.

#### Emotional support

There was a significant decline over time in emotional support for all women, as indicated from the significant coefficients of time in Model 1 and the main effects of time in Model 2 (Table 2). There was no significant difference between the two groups for the first three time points, but at 4 years the difference in perceived emotional support between the two groups increased, indicated by the significant interaction term at 4 years postpartum in Model 2. Figure 1 shows this non-linear trend, with perceived emotional support for women without MDD beginning to level off after 12 months postpartum, but continuing to decline for women with MDD up to 4 years postpartum.

Significant covariates for declining perceived emotional support were ongoing depressive symptoms, number of stressful life events and maternal anxious or avoidant attachment orientation in close relationships.

#### Support with tasks and responsibilities

For support with tasks and responsibilities, there was also a significant decline over time for both women with and without MDD, as indicated from the significant coefficients of time in Model 1 and the main effects of time in Model 2 (Table 2). For women with MDD, perceptions of support for tasks and responsibilities declined further at 12 months postpartum and remained lower at 4 years, as indicated by the significant interaction terms at these time points (Table 2, Model 2). For women without MDD, perceived task support levelled off after 6 months postpartum, as shown in Fig. 1. Again, the trend of perceived support with tasks and responsibilities over time was non-linear.

Significant covariates for declining support with tasks and responsibilities were ongoing depressive symptoms, number of stressful life events, and maternal anxious and avoidant attachment orientation in close relationships.

#### Overall support

The results for perception of overall support were similar, with a significant decline overall in perceptions of partner support for both women with and without MDD. The two groups only differed at 4 years postpartum, with lower perceptions of overall support for women with MDD.

Ongoing symptoms of depression and distress were significantly associated with lower perceptions of overall support, even after diagnosis of MDD was controlled for. Other significant covariates of decline in overall support were number of ongoing stressful life events and maternal anxious or avoidant attachment orientation in close relationships.

#### Interpretation of random effects parameters

The random intercepts of all models had significant variances, as indicated by their confidence intervals (Table 2). This indicates that the perceived partner support baseline scores at recruitment varied significantly between individuals. Some of the variances of the random time effects were also significantly above zero, suggesting that the effects of time may vary between individuals. This confirms that the use of mixed models with random effects for the intercept and for time is appropriate for this data, allowing for individual variation.

## Discussion

This study was unique in focusing on the longitudinal pattern of the perceived effectiveness of two different types of partner support in women with and without a diagnosis of MDD. We found a small but significant decrease in perceived effectiveness of partner support over time for all women. For women without MDD this decline flattened off after 6 months postpartum for emotional support and 12 months postpartum for task support. However, for women with MDD there was a longer-term decline until 4 years postpartum. Ongoing depressive symptoms, stressful life events, and insecure attachment orientation all contributed to a perception of lower support effectiveness for all women. These results suggest that the perinatal and early childhood period poses a small increased risk to the partner relationship for all women, but this risk is higher for women with MDD. The findings of decreased perceived support over time are consistent with many existing studies indicating a relationship decline postpartum (Kluwer 2010; Mitnick et al. 2009) and in early childhood ( Kohn et al. 2012). Nevertheless, some large studies have found stable relationship satisfaction trajectory over time for the majority of women (Kingsbury et al. 2023). Kingsbury et al. assessed overall relationship satisfaction, whereas our study examined specific aspects of partner support, which makes it difficult to compare the two studies directly. However, Kingsbury et al. found that for a small group of women (7%) who reported declining relationship satisfaction from pregnancy to postpartum this was associated with a history of depression, including postnatal depression. This is consistent with our findings that MDD presents a risk for the partner relationship.

The contribution of both ongoing distress and an insecure attachment pattern to the decline in perceived partner support effectiveness was consistent with previous literature (Kohn et al. 2012; Leonhardt et al. 2022; Rholes et al. 2014). We did not find significant effects for demographic and socioeconomic factors, as some previous studies have (Hetherington et al. 2020; Keizer et al. 2010; Nakamura et al. 2020).

The steepest decline in perceived support for all women occurred in the months after the birth. This often coincides with the partner returning to work with less available support and high mothering demands. This is combined with increased household labour and responsibilities for the baby, as well as sleep deprivation and fatigue (Kluwer 2010). At the same time women’s social networks narrow and the importance of partner support increases (Kroelinger and Oths 2000; Pajulo et al. 2001; Rini 2001). In the longer term, as the child gets older, women may also need to juggle more roles and responsibilities, including returning to employment and other children in the family. These stresses may have more impact on women with MDD and in the long term increase the discrepancy between the support needed and support provided.

The results for emotional and practical support were similar, with both continuing to decline over time for women with MDD. However, practical support was lower for women with MDD than for women without MDD at both 6 months and 4 years postpartum, whereas emotional support was only lower at 4 years postpartum. Perhaps at around 6 months postpartum when the partner is likely to return to work the loss of practical support is more immediately salient for women with MDD who may become more overwhelmed with tasks. Support with specific tasks and responsibilities may be more tangible and more visible, especially at the early postpartum stage. In contrast, emotional support may be more complex to provide and receive, requiring skills such as communicating, listening and validating (Morelli et al. 2015; Shrout et al. 2006). Therefore, it is important to examine the different types of support separately.

Depression may affect women’s perceptions of support by increasing negative cognitions associated with evaluating others more negatively and perceptions of being treated more negatively by others (Ibarra-Rovillard and Kuiper 2011). Consequently, negatively biased cognitive appraisals will be reflected in self-report questionnaires of partner support.

Since perceived emotional and practical support are potentially modifiable protective factors (Pilkington et al. 2015a, b), our findings have a clinical significance. The perinatal period is a time when women are more frequently in contact with health services, providing the opportunity for identifying risk factors and offering interventions. It is important for health professionals to regularly assess the quality of the partner relationship throughout the perinatal period and offer appropriate interventions to strengthen the relationship. Although the differences between women with and without MDD were small, they were long lasting. Therefore, it is important to provide targeted mental support for women with MDD which includes involving the partner, perhaps in the form of psychoeducation and practical relationship and support skills training. Online support, such as the SMS4Dads (Fletcher et al. 2025; Lanning et al. 2022) program for partners of mothers with a major mental illness, shows promising evidence. Availability of parental leave for fathers is another significant factor associated with higher relationship quality postpartum (e.g. Petts and Knoester 2019). It is also important to offer intervention programs and parental support in the longer term, not just the early months postpartum. Pilkington et al. (2015a, b) found in a systematic review that key factors which can be targeted to reduce perinatal depression are: positive communication, emotional closeness, emotional support, satisfaction with the division of household labour following childbirth, instrumental/practical support, and global support. However, systematic reviews show inconclusive evidence for the effectiveness of existing partner-inclusive perinatal mental health prevention (Pilkingtonet al., 2015a) and intervention (Alves et al. 2018) programs which have included a partner component, with low attendance rate being a major limitation. Our findings also suggest that clinical assessments and interventions may need to consider different aspects of partner support, such as emotional and practical, and how to specifically target each type of support. This underlines the importance of further research in this area, including for programs targeted for women with MDD.

It is important to note that our study focused on women with a continuing relationship, and therefore future research needs to establish to what extent the results can be generalised to women without a continuing relationship over a long period of time. Our results showed that women without a continuing relationship were more likely to be diagnosed with MDD at recruitment. It is likely that they may have faced more relationship challenges and perhaps experienced a more negative change in partner support. A previous study found that mothers with lower initial relationship satisfaction experienced steeper relationship satisfaction decline from pregnancy to 9 months postpartum than those with higher initial satisfaction (Don and Mickelson 2014). However, for some women a relationship with a different partner may result in increased support.

### Strengths

This study makes an important and unique contribution to a better understanding of the changes in perceived partner support over the perinatal period and early childhood by focusing on women with diagnosed MDD and specific types of partner support, for which the existing literature is scarce. The use of the SCID, a gold standard diagnostic tool, to diagnose MDD is an important strength of the study. The longitudinal design is also a strength of the study. Moreover, this study included a deliberate oversampling of women with MDD, thus providing an adequate sample size to focus on this group. 

### Limitations

The study did not collect data from the partner, thus we were not able to explore the couples’ reciprocal perceptions of support. There was no baseline data on partner support pre-pregnancy or during early pregnancy, although some studies indicate improvement in relationship satisfaction during pregnancy (Kluwer 2010). The spacing of time points with a gap between 12 months and 4 years postpartum, meant that we were not able to determine if there were changes during this period. Nevertheless, our results of overall decline during this period are consistent with other studies (e.g. Kohn et al. 2012). Symptoms of distress and partner support were measured by self-report questionnaires. However, evidence indicates that self-reported perceived support is a more useful measure than received support measured by observed quantity, as it has a stronger association with mental health (Lakey and Cronin 2008; Uchino 2009). The significant variances of the random intercept and the random effects at some time points suggest that there may be subgroups of women with different pattern of changes of partner support over time. However, it was not feasible to investigate this in the current study due to insufficient sample size. It was also not feasible to examine if the effects of the significant covariates differed for women with and without MDD due to insufficient sample size. Our study only included women in a continuing relationship with the same partner from first trimester to 4 years postpartum, and it is not known to what extent these results are generalisable to women with different kinds of relationships.

## Conclusions

This study found an overall small but significant decline in partner support over time for all women. However, this decline was larger for women with MDD between 12 months and 4 years postpartum. Ongoing depressive symptoms, stressful life events, and insecure attachment style also contributed to perceptions of lower partner support. The perinatal and early childhood period poses an increased risk for the partner relationship for all women, but there is an increased risk for women with MDD. This knowledge could be translated into identifying women at higher risk of lower partner support and offering appropriate interventions. To examine the generalisability of our results, future research should specifically focus on women from various backgrounds, including socioeconomically disadvantaged, women without a continuing relationship or women without a partner, and same sex couples. 

## Data Availability

No datasets were generated or analysed during the current study.
